# Multiple alignment comparison of the non-structural genes of three strains of equine influenza viruses (H3N8) isolated in Morocco

**DOI:** 10.1186/s13104-015-1441-0

**Published:** 2015-09-24

**Authors:** Mohamed Boukharta, Souad Azlmat, Mehdi Elharrak, My Mustapha Ennaji

**Affiliations:** Laboratory of Virology, Microbiology and Quality/ETB, Faculty of Sciences and Techniques, Mohammedia, University Hassan II Mohammedia-Casablanca, PO BOX 146, Quartier Yasmina, Mohammedia, 20650 Morocco; Department of Biology, Instruction Military Hospital Med V Rabat, University Mohammed V Souissi, Rabat, Morocco; Society of Pharmaceutical and Veterinary Products, Virology Laboratory, Av Hassan II, BP 4569 Rabat, Morocco

**Keywords:** Equine influenza virus, Non-structural protein (NS), RNA-binding domain, Effector domain

## Abstract

**Background:**

Three equine influenza viruses, A/equine/Nador/1/1997(H3N8), A/equine/Essaouira/2/2004(H3N8), and A/equine/Essaouira/3/2004(H3N8), were isolated from different Equidae during local respiratory disease outbreaks in Morocco in 1997 and 2004. Their non-structural (NS) genes were amplified and sequenced.

**Results:**

The results show high homology of NS nucleotide sequences of A/equine/Nador/1/1997 with European strains (i.e., A/equine/newmarket/2/93 and A/equine/Grobois/1/1998) and clustered into the European lineage. However, NS gene of A/equine/Essaouira/2/2004(H3N8) and A/equine/Essaouira/3/2004(H3N8) strains indicated high homology with equine influenza strains that had circulated before 1990 (A/equine/Fontainbleu/1/1979(H3N8), which belonged to a pre-divergent phase Amino acid sequence comparison of the NS1 protein with reference strain A/equine/Miami/1963(H3N8) shows that the A/equine/Nador/1/1997(H3N8) strain has 12 substitutions at the residues D/24/N, R/44/K, S/48/I, R/67/Q, A/86/V, E/139/K, A/112/T, E/186/K, L/185/F, A/223/E, S/213/T and S/228/P. In both A/equine/Essaouira/2/2004(H3N8) and A/equine/Essaouira/3/2004(H3N8) strains, the NS1 sequences present one common mutation at the residue: S/228/P.

**Conclusion:**

It seems that all of these substitutions are not produced at the key residues of the RNA-binding domain (RBD) and the effector domain (ED). Consequently, we can suppose that they will not affect the potency of inhibition of cellular defences, and the virulence of the Moroccan equine strains will be maintained.

## Background

Equine influenza virus is a highly contagious agent, capable of causing explosive outbreaks of respiratory disease among susceptible horse populations in many countries [1]. Two distinct subtypes of influenza virus, H7N7 (prototype influenza A/equine/Prague/1/56) and H3N8 (prototype influenza A/equine/Miami/1/63), have been recognized in the horse [2, 3]. The first subtype (H7N7) is believed to be extinct and has not been isolated in the horse since 1979. The other subtype (H3N8) was isolated for the first time in 1963 and was responsible for all recent equine influenza outbreaks [4, 5]. These viruses belong to the Orthomyxoviridae family type A. The genome comprises eight segments of RNA [6]. These segments are encapsulated by a nucleoprotein (NP), which gives a helical symmetry to each. Six segments encode one protein: hemagglutinin (HA), neuraminidase (NA), nucleoprotein (NP), and three polymerases: PA, PB1, and PB2. Each of the other segments encodes two proteins: the matrix (M1 and M2) and a non-structural protein (NS1) and NS2 (NEP: nuclear export). The genomes of several virulent strains encode an 11th protein PB1-F2 [7]. NS1, NS2, and PB1-F2 proteins participate in viral replication and are not incorporated into the viral structure.

The presence of double-stranded RNA (dsRNA) is a signal of a viral infection, causing the activation of the cellular defence system through the synthesis of the interferon protein (IFN) [8]. However, influenza viruses have developed a strategy to antagonize the cellular defences. The non-structural protein NS1 is regarded as the major antagonist of the immune response of the host cells [9]. In most viruses, the NS1 protein consists of 230 amino acids (aa) and the NEP protein of 121 aa [10]. However, a size variability of the NS1 protein was observed, especially in the human and swine viruses [11]. In the case of H3N8 equine influenza virus, truncations have been shown as well as other influenza viruses [12, 13].

The linear structure of the NS1 protein contains two domains linked by a non-structured region composed of ten amino-acids: The first one is the RNA-binding domain (RBD), representing the N-terminal protein (amino acids 1–73), the second is an effector domain (ED), which represents C-terminal protein (amino acids 74–230) [14].

The RNA-binding domain has an alpha-helical structure and forms a symmetrical homo-dimer with a unique six-helical chain fold. The effector domain forms a dimer, and each monomer consists of seven beta-strands and three alpha helices [15]. The non-structural protein NS1 contains several interaction domains: the region of binding to dsRNA (residues 1–73), the specific cleavage and polyadenylation specific factor region (CPSF) (residues 175–210), the region of the binding protein (PABP)—II (poly (A)—binding protein—II) (residues 218–225), the region of nuclear localization signal 1 (NLS 1) (residues 34–38), the region nuclear localization signal 2 (NLS 2: nuclear localization signal) (residues 211–216), the region of the nuclear export signal (NES) (residues 132–141), and the region that interacts with subunit regulatory p85-β of phosphatidylinositol 3-kinase (PI3K) (residues 89–93, 137–142 and 164–167) [16].

The PDZ domain binding is present at the C-terminal of NS1 among equine influenza viruses (H3N8) (sequence ESEV/EPEV), and it is known as a determinant of viral virulence.

In the present study, we report analysis of the partial nucleotide sequence of the NS gene and the NS1 protein for three Moroccan equine influenza viruses: A/Equine/Nador/1/97, A/Equine/Essaouira/2/2004, and A/Equine/Essaouira/3/2004, and their genetic comparison with influenza strains (human, equine, swine, avian) available in the GenBank database.

## Methods

### Viruses

A/equine/Nador/1/97 was isolated in Nador from a mule after four passages on eleven -day-old embryonated specific pathogen-free chicken eggs (ECE4) as described by Kissi et al. (1998) [17]. Both A/equine/Essaouira/2/2004 and A/equine/Essaouira/3/2004 were isolated respectively from an infected donkey and a horse during 2004 outbreaks in Essaouira. The isolates were passaged on Madin Darby Canine Kidney cell line after two passages (MDCK2) at 34 °C in an atmosphere of 5 % CO_2_ in Eagle’s minimum essential media supplemented with 5 % foetal calf serum.

### Viral RNA extraction and amplification

Viral RNA was extracted directly from isolates using a PureLink Viral RNA/DNA Mini-Kit (Invitrogen, Van Allen Way, Carlsbad, California, USA) following the manufacturer’s recommended protocol. Complementary DNA was obtained by RT reactions, which were carried out by using a SuperScript III First-Strand Synthesis System (Life Technologies, Carlsbad, CA, USA).

PCR was performed using a Platinum PCR SuperMix High-Fidelity Kit (Invitrogen, Carlsbad, CA, USA) with cDNA obtained using primers specific for NS1F (*ATGGATTCCAACACTGTGTC*) and NS1R (*TCAAACTTCTG(A/G)CTCAATTG*) at a final concentration of 0.5 µM for primers. Primer design is detailed by Tissier (2008) [18] and was synthesized by the Unité d’Appui Technique à la Recherche Scientifique, Centre National de Recherche Scientifique et Technique (CNRST), Rabat, Morocco. The assay was performed on the SmartCycler instrument (Cepheid, Sunnyvale, CA, USA), using the following thermocycling protocol: incubation at 95 °C for 2 min, then 35 cycles of denaturation at 95 °C for 30 s, 52 °C for 1 min for hybridization, and 72 °C for 30 s, and a final extension for 3 min at 72 °C.

### Sequencing NS Genes and phylogenetic Analysis

The amplified PCR NS products were sequenced. Briefly, the PCR products were purified using EXOSAP-IT (USB Corporation, Cleveland, OH, USA) and bi-directionally sequenced by using BigDye1 Terminator v3.1 (Applied Biosystems, Foster City, Calif. USA) on a 3130xl model sequencer (Applied Biosystems). Analysis of the electro-phoregramm was carried out with the sequencing analysis software version 5.3.1 (Applied Biosystems). We performed phylogenetic analysis of 54 influenza strains (including equine, avian and human isolates) published in GenBank database, selected using the neighbour-joining method with bootstrap analyses of 500 replicates in CLUSTAL W. The tree was visualized using MEGA5.1 software (http://megasoftware.net/) [19].

### Determination of amino acid sequences of NS genes

Multiple alignments of the deduced amino acid sequences were used by Basic Local Alignment Search Tool (BLAST) software. Comparing NS1 protein of Moroccan isolates to the reference strain (A/Equine/Miami/1/1963), the amino acid substitutions that were involved in both *RNA*-*binding domain* (RBD) and *effector domain* (ED) were determined.

## Results and discussion

Partial NS nucleotide sequences of the Moroccan equine influenza isolates (A/equine/Nador/1/1997, A/equine/Essaouira/2/2004, and A/equine/Essaouira/3/2004) were submitted to the GenBank database, and their accession numbers of NS genes are JX182368, JQ955611, and JQ955614, respectively.

### BLAST results

The BLAST result shows that the nucleotide sequences of the NS gene of two strains A/equine/Essaouira/2/2004 and A/equine/Essaouira/3/2004 presents high similarity between them, and towards other old strains like A/equine/Miami/1/1963 (>99 %), A/equine/Uruguay/1/63(99 %), A/eq/LaPlata/1/88 (>99 %), and A/equine/Fontainebleau/79 (98 %). These strains have circulated before 1990. Since then, the equine influenza viruses have diverged into two antigenically distinct lineages, Eurasian and American. According to several authors, the pre-divergent strains have disappeared and were supplanted by viruses that evolved in lineages and sub-lineages [20].

The partial nucleotide sequence of the NS gene of A/equine/Nador/1/97 strain is genetically distant towards the pre-divergent strains. Indeed, it demonstrates high homology to the strains that have circulated in Europe during the 1990s, i.e., A/equine/Newmarket/2/1993 (99 %), A/equine/Avesta/1/1993 (99 %), A/equine/Grobois/1/98 (98 %), A/equine/Italy/1199/1992 (98 %), and A/equine/Roma/5/1991 (98 %).

### Phylogenetic analysis

**Fig. 1:**
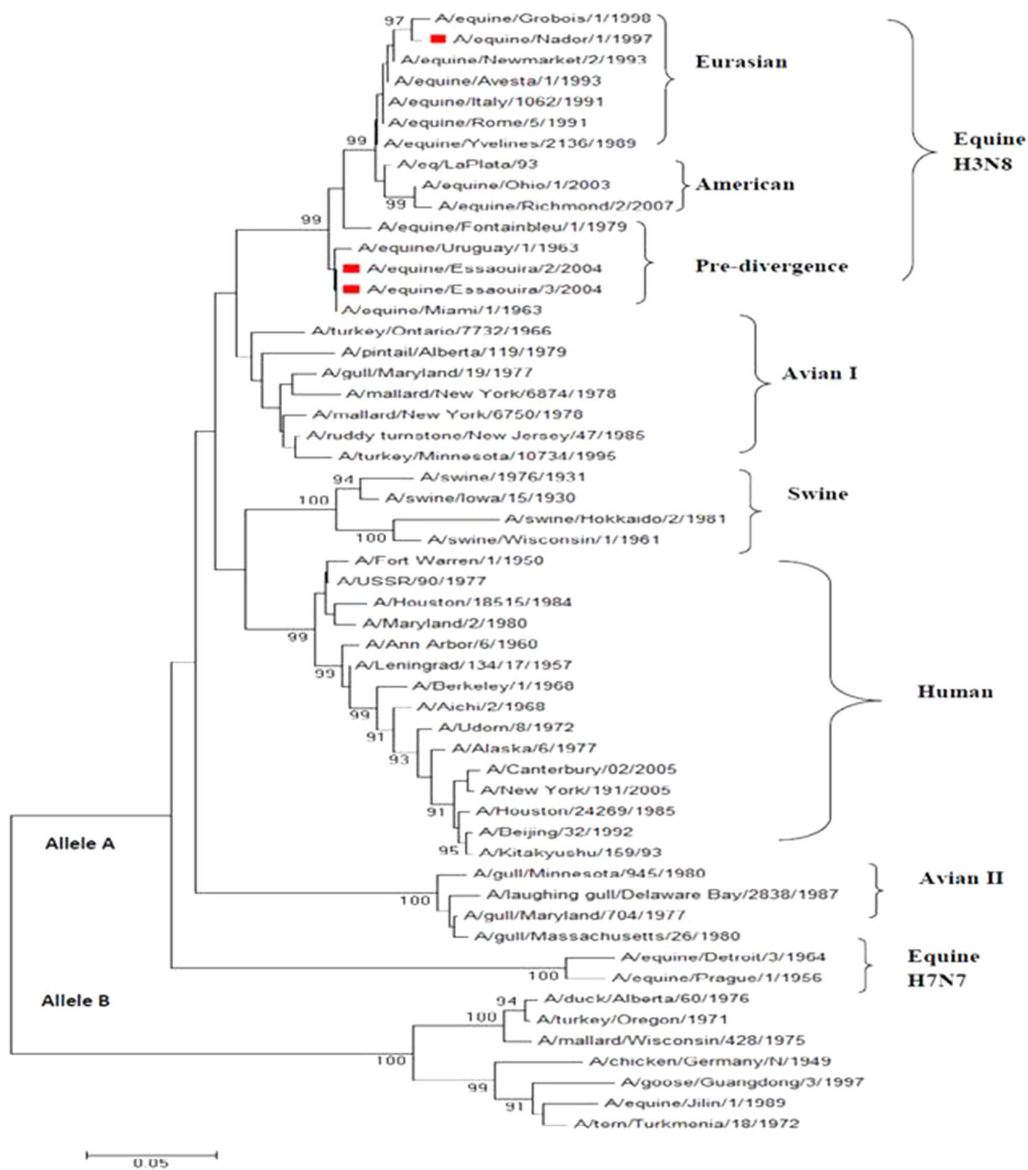
Phylogenetic analysis of the partial nucleotide sequences of NS of 54 strains (human, avian, swine and equine). A/Aichi/2/1968(H3N2), *M34829*; A/Alaska/6/1977(H3N2), *K01332*; A/AnnArbor/6/1960(H2N2), *M23968*; A/Beijing/32/1992(H3N2), *D30667*; A/Berkeley/1/1968(H2N2), *M12590*; A/Canterbury/02/2005(H3N2), *CY007807*; A/FortWarren/1/1950(H1N1), *K00576*; A/Houston/18515/1984(H1N1), *M12594*; A/Houston/24269/1985(H3N2), *M17699*; A/Kitakyushu/159/93(H3N2), *D30676*; A/Leningrad/134/17/1957(H2N2), *M81578*; A/Maryland/2/1980(H1N1), *M12595*; A/NewYork/191/2005(H3N2), *CY006127*; A/USSR/90/1977(H1N1), *K00578*; A/Adorn/8/1972(H3N2), *V01102*; A/chicken/Germany/N/1949(H10N7), *AF001407*; A/duck/Alberta/60/1976(H12N5), *J02105*; A/eq/LaPlata/93(H3N8), *AF001673*; A/equine/Avesta/1/1993(H3N8), *AB543512*; A/equine/Detroit/3/1964(H7N7), *M80970*; A/equine/Essaouira/2/2004(H3N8), *JQ955611*; A/equine/Essaouira/3/2004(H3N8), *JQ955614*; A/equine/Fontainbleu/1/1979(H3N8), *CY032409*; A/equine/Grobois/1/1998(H3N8), *AY328471*; A/equine/Italy/1062/1991(H3N8), *CY032377*; A/equine/Jilin/1/1989(H3N8), *M65020*; A/equine/Miami/1/1963(H3N8), *CY028840*; A/equine/Nador/1/1997(H3N8), *JX182368*; A/equine/Newmarket/2/1993(H3N8), *FJ375211*; A/equine/Ohio/1/2003(H3N8), *DQ124186*; A/equine/Prague/1/1956(H7N7), *M80944*; A/equine/Richmond/2/2007(H3N8), *FJ195442*; A/equine/Rome/5/1991(H3N8), *AF001669*; A/equine/Uruguay/1/1963(H3N8), *CY032425*; A/equine/Yvelines/2136/1989(H3N8), *AF001666*; A/goose/Guangdong/3/1997(H5N1), *AY028445*; A/gull/Maryland/19/1977(H2N9), *CY005810*; A/gull/Maryland/704/1977(H13N6), *M80959*; A/gull/Massachusetts/26/1980(H13N6), *U96744*; A/gull/Minnesota/945/1980(H13N6), *U96738*; A/laughing gull/Delaware Bay/2838/1987(H13N2), *CY005068*; A/mallard/New York/6750/1978(H2N2), *M80945*; A/mallard/NewYork/6874/1978(H3N2), *M25375*; A/mallard/Wisconsin/428/1975(H5N1), *U85380*; A/pintail/Alberta/119/1979(H4N6), *M25374*; A/ruddy turnstone/New Jersey/47/1985(H4N6), *M80946*; A/swine/1976/1931(H1N1), *M55482*; A/swine/Hokkaido/2/1981(H1N1), *M80961*; A/swine/Iowa/15/1930(H1N1), *M80965*; A/swine/Wisconsin/1/1961(H1N1), *M80951*; A/tern/Turkmen/18/1972(H3N3), *M55466*; A/turkey/Minnesota/10734/1995(H5N2), *U85391*; A/turkey/Ontario/7732/1966(H5N9), *U85376*; A/turkey/Oregon/1971(H7N3), *M16623*. Numbers next to nodes indicate bootstrap value percentages (>90 %)

Data obtained from BLAST was confirmed by the phylogenetic analysis based on 54 nucleotide sequences of the NS gene of influenza A viruses, which were isolated from various species (human, avian, swine, and equine). The NS genes of the Moroccan strains are clustered into a lineage of the equine influenza virus (H3N8) that belongs to the A allele. The two strains, A/equine/Essaouira/2/2004 and A/equine/Essaouira/3/2004, belong to the pre-divergent phase, while the A/equine/Nador/1/97 belongs to the Eurasian lineage (Fig. 1). Moreover, the sequencing analysis of the HA (accession numbers JQ955607, JQ955609, and JQ955612) of A/equine/Nador/1/1997, A/equine/Essaouira/2/2004, and A/equine/Essaouira/3/2004, respectively) revealed same data. Such results show that these three strains isolated in Morocco share the same ancestors as the equine influenza (H3N8) virus and that there was no genetic reassortment between co-circulating strains in Morocco [21].

The data matrix containing 54 taxa and phylogenetic analyses were deposited into TreeBase under accession url: http://purl.org/phylo/treebase/phylows/study/TB2:S18066.

### Comparison analysis of amino acids alignment

**Fig. 2:**
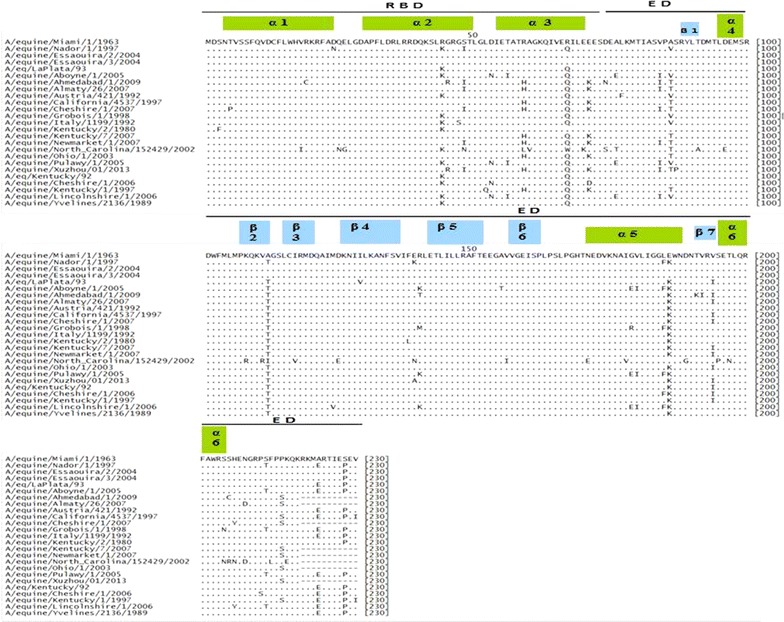
Amino acid alignment of 25 predicted NS sequences compared to A/equine/Miami/1963. A/equine/Miami/1/1963, *ABY81497*; A/equine/Nador/1/1997, *AFN69204*; A/equine/Essaouira/2/2004, *AFJ69907*; A/equine/Essaouira/3/2004, *AFJ69911*; A/eq/LaPlata/93, *AAC31270*; A/equine/Aboyne/1/2005, *ACH95651*; A/equine/Ahmedabad/1/2009, *ADB45168*; A/equine/Almaty/26/2007, *ADE21678*; A/equine/Austria/421/1992, *ACD85324*; A/equine/California/4537/1997, *ACA96805*; A/equine/Cheshire/1/2007, *ACH95645*; A/equine/Grobois/1/1998, *AAP92165*; A/equine/Italy/1199/1992, *ACD85313*; A/equine/Kentucky/2/1980, *ACF22120*; A/equine/Kentucky/7/2007, *ACH95629*; A/equine/Newmarket/1/2007, *ACH95607*; A/equine/North Carolina/152429/2002, *ACZ47169*; A/equine/Ohio/1/2003, *ABA42431*; A/equine/Pulawy/1/2005, *AIW60784*; A/equine/Xuzhou/01/2013, *AHA98359*; A/eq/Kentucky/92, *AAC31266*; A/equine/Cheshire/1/2006, *ACH95605*; A/equine/Kentucky/1/1997, *ACH95653*; A/equine/Lincolnshire/1/2006, *ACH95599*; A/equine/Yvelines/2136/1989, *AAC31256*. Topology of α-helix and β-strands of NS1 protein: α1(4–24), α2 (31–50), α3 (54–70), α4(95–99), α5(171–188), α6 (195–205), β1(88–91), β2 (107–112), β3 (115–120), β4(126–136), β5(142–151), β6 (157–162), β7 (191–194) [15]

Compared to the reference strain A/equine/Miami/1/1963, the sequences of amino acids of the NS1 protein of the strain A/equine/Nador/1/1997 have 12 substitutions: D/24/N, R/44/K, S/48/I, R/67/Q, A/86/V, E/139/K, A/112/T, E/186/K, L/185/F, A/223/E. S/213/T, and S/228/P. These mutations occurred in both RNA-binding and effector domains (Fig. 2).

The D/24/N substitution occurred in a changing region of the RBD (19 to 28) of the NS1 protein of influenza viruses [22]. This mutation may not affect the structure of the NS1 protein RNA binding domain or its activity. This finding is confirmed by the virulence of the A/equine/Nador/1/1997 strain, which caused severe respiratory signs among naturally infected mules. The same substitution was also reported by Parveen and Asad (2010) [23] concerning the human strain A/North Dakota/04/2009 (H1N1) (ACR67137).

The three mutations (R/44/K, S/48/I, and R/67/Q) revealed on the RBD do not seem to affect virulence, because they do not concern the following residues: Thr-5, Pro-31, Asp-34, Arg-35, Arg-38, Lys-41, Gly-45, Arg-46, and Thr-49, which are closely linked to the activity of the NS1 protein. Several authors have reported that these nine amino acids are the key residues of RBD activity [9, 10, 24].

Mutations that occurred on the effector domain were reported in several sites recognized as having functional roles. In fact, mutations at residues A/86/V and A/112/T occurred in a specific region of the eIF4GI protein (81 to 113), and they participate with the protein PABPI in the initiation of mRNA viral translation [25, 26].

The binding of the protein CPSF30 (cleavage and polyadenylation specific factor) inhibits the maturation of cellular pre-mRNA. This link is provided by amino acids 144, 184, and 188 (186 region) [15, 27]. It appears that both L/185/F and E/186/K mutations may not reduce the affinity of NS1 to CPSF30 protein, and consequently do not inhibit viral replication.

Three substitutions, E/139/K, S/213/T, and A/223/E, were reported in domain activity of nucleocytoplasmic distribution in the cell of the NS1 protein. The E/139/K mutation is located at the nuclear export signal region (NES: nuclear export signal) (residues 132–141) [16]. Furthermore, similar mutations at this residue were also observed for the NS1 of the following strains: N/139/E (A/equine/North Carolina/152429/2002, ACZ47169); and E/139/A (A/equine/Xuzhou/01/2013, AHA98359) (Fig. 2). The two mutations, S/213/T and A/223/E, occurred at nuclear localization signal (NLS2) and PABPII domains, respectively. NLS2 is defined within the region between amino acids 213 and 221. This domain is present in the NS1 proteins of most influenza A virus strains. However, several substitutions affecting it were noticed, such as: S/213/T (i.e.,: A/equine/Nador/1/1997, AFN69204 and A/equine/Aboyne/1/2005, ACH95651), and P/214/S (i.e.,: A/equine/Cheshire/1/2007, ACH95645). However, nuclear localization signal 1 (NLS1) contains the stretch of basic amino acids Asp-Arg-Leu-Arg-Arg (codons 34 to 38). This sequence is conserved in three Moroccan strains as all NS1 proteins of influenza A viruses [28]. It appears that the substitution occurred in NLS2 do not seem to interfere with the nuclear localization of NS1 protein; because the intracellular localization is regulated by one or two nuclear localization signals (NLS) and a nuclear export signal (NES) [29].

Multiple comparisons of the NS1 protein of 27 equine influenza viruses show that the mutations D/24/N and E/139/K characterize the A/equine/Nador/1/1997(H3N8) strain, while the A/86/V and S/213/T mutations are shared with several European lineage strains. However, the eight substitutions (R/44/K, S/48/I, R/67/Q, A/112/T, E/186/K, L/185/F, A/223/E, and S/228/P) are present in various equine influenza viruses (H3N8) belonging to different phases of evolution (pre-divergence and the American and European lineages) (Table 1).

**Table 1:** Mutations comparison of non-structural protein (NS1) of 27 equine influenza viruses

Strains	Amino-acid positions																				
	24	33	44	47	48	53	56	59	67	69	71	77	79	81	84	86	91	96	98	111	112
A/equine/Miami/1/1963	D	L	R	G	S	D	T	R	R	L	E	L	M	I	V	A	T	D	M	V	A
A/Equine/Nador/1/1997	N	.	K	.	I	.	.	.	Q	.	.	.	.	.	.	V	.	.	.	.	T
A/Equine/Essaouira/2//2004	.	.	.	.	.	.	.	.	.	.	.	.	.	.	.	.	.	.	.	.	.
A/Equine/Essaouira/3/2004	.	.	.	.	.	.	.	.	.	.	.	.	.	.	.	.	.	.	.	.	.
A/equine/Uruguay/1/1963	.	.	.	.	.	.	.	H	.	.	.	.	.	.	.	.	.	.	I	.	.
A/equine/Algiers/1/1972	.	.	K	.	.	.	.	.	G	.	.	.	R	.	.	.	I	E	.	.	.
A/equine/Romania/1/1980	.	.	K	.	.	.	.	.	.	.	.	.	.	T	.	.	.	.	.	.	T
A/equine/Fontainebleau/1979	.	.	K	.	.	.	.	.	.	.	.	.	.	.	.	.	.	.	.	.	T
A/equine 2/Suffolk/89	.	.	K	.	.	.	.	.	Q	.	.	F	.	.	.	.	.	.	.	.	T
A/equine/Yvelines/2136/1989	.	.	K	.	.	.	.	.	Q	.	.	.	.	.	.	.	.	.	.	.	T
A/equine/Newmarket/2/1993	.	.	K	.	.	.	.	.	Q	.	.	.	.	.	.	V	.	.	.	.	T
A/equine/Italy/1199/1992	.	.	K	S	.	.	.	.	Q	.	.	.	.	.	.	V	.	.	.	.	T
A/eq/Roma/5/91	.	.	K	.	.	.	.	.	Q	M	.	.	.	.	.	V	.	.	.	.	T
A/equine/Avesta/1/1993	.	.	K	.	.	.	.	.	Q	.	.	.	.	.	.	V	.	.	.	.	T
A/equine/Berlin/1/1989	.	.	K	.	.	.	.	.	Q	.	.	.	.	.	.	.	.	.	.	.	T
A/equine/Grobois/1/1998	.	.	K	.	.	.	.	.	Q	.	.	.	.	.	.	V	.	.	.	.	T
A/eq/LaPlata/93	.	.	K	.	.	.	.	.	Q	.	.	.	.	.	.	.	.	.	.	.	T
A/equine/Florida/2/2006	.	.	.	.	.	.	.	H	Q	.	K	.	.	.	.	T	.	.	.	I	T
A/equine/Ohio/1/2003	.	.	.	.	.	.	.	H	Q	.	K	.	.	.	.	T	.	.	.	.	T
A/equine/Richmond/1/2007	.	.	.	.	I	.	.	H	Q	.	K	.	.	.	I	T	.	.	.	.	T
A/equine/Kentucky/7/2007	.	.	.	.	.	.	.	H	Q	.	K	.	.	.	.	T	.	.	.	.	T
A/equine/Wisconsin/1/03	.	.	.	.	.	.	.	H	Q	.	K	.	.	.	.	T	.	.	.	.	T
A/equine/California/1/2007	.	.	.	.	.	.	.	H	Q	.	K	.	.	.	.	T	.	.	.	.	T
A/equine/Cheshire/1/2006	.	.	K	.	.	N	.	.	Q	.	D	.	.	.	.	.	.	.	.	.	T
A/equine/Kentucky/1/1992	.	.	K	.	.	.	.	.	Q	.	.	.	.	.	.	.	.	.	.	.	T
A/equine/Newmarket/1/1993	.	.	K	.	.	N	.	.	Q	.	.	.	.	.	.	.	.	.	.	.	T
A/equine/Newmarket/5/2003	.	.	.	.	I	.	.	H	Q	.	K	.	.	.	I	T	.	.	.	.	T

Strains	Amino-acid positions																				
	117	129	139	140	146	179	185	186	194	197	205	207	209	212	213	216	223	227	228	229	230
A/equine/Miami/1/1963	I	I	E	R	L	G	L	E	V	T	S	H	N	P	S	P	A	E	S	E	V
A/Equine/Nador/1/1997	.	.	K	.	.	.	F	K	.	.	.	.	.	.	T	.	E	.	P	.	.
A/Equine/Essaouira/2//2004	.	.	.	.	.	.	.	.	.	.	.	.	.	.	.	.	.	.	P	.	.
A/Equine/Essaouira/3/2004	.	.	.	.	.	.	.	.	.	.	.	.	.	.	.	.	.	.	P	.	.
A/equine/Uruguay/1/1963	.	.	.	.	.	.	.	.	.	.	.	.	D	.	.	.	.	.	.	.	.
A/equine/Algiers/1/1972	.	.	.	.	.	E	.	.	.	.	.	.	.	.	.	S	–	–	–	–	–
A/equine/Romania/1/1980	.	.	.	.	.	.	.	K	.	A	.	N	.	.	.	.	.	.	.	.	.
A/equine/Fontainebleau/1979	V	.	.	.	I	.	.	K	.	A	.	N	.	.	.	.	.	.	.	.	.
A/equine 2/Suffolk/89	.	.	.	.	.	.	.	K	.	.	.	.	.	.	.	.	E	.	.	.	.
A/equine/Yvelines/2136/1989	.	.	.	.	.	.	.	K	.	.	.	.	.	.	.	.	E	.	P	.	.
A/equine/Newmarket/2/1993	.	.	.	.	.	.	.	K	.	.	.	.	.	.	T	.	E	.	P	.	.
A/equine/Italy/1199/1992	.	.	.	.	.	.	.	K	.	.	.	.	.	.	.	.	E	.	P	.	.
A/eq/Roma/5/91	.	.	.	.	.	.	.	K	.	.	.	.	.	.	.	.	E	.	P	.	.
A/equine/Avesta/1/1993	.	.	.	.	.	.	.	K	.	.	.	.	.	.	.	.	E	.	P	.	.
A/equine/Berlin/1/1989	.	.	.	.	.	.	.	K	.	.	.	.	.	.	.	.	E	.	P	.	.
A/equine/Grobois/1/1998	.	.	.	M	.	R	F	K	.	.	N	.	.	.	T	.	E	.	P	.	.
A/eq/LaPlata/93	.	V	.	.	.	.	.	K	I	.	.	.	.	.	.	.	E	.	P	.	.
A/equine/Florida/2/2006	.	.	.	.	.	.	.	K	I	.	.	.	.	.	.	S	–	–	–	–	–
A/equine/Ohio/1/2003	.	.	.	.	.	.	.	K	I	.	.	.	.	.	.	S	–	–	–	–	–
A/equine/Richmond/1/2007	.	.	.	.	.	.	.	K	I	.	.	Y	.	.	.	S	–	–	–	–	–
A/equine/Kentucky/7/2007	.	.	.	.	.	.	.	K	I	.	.	.	.	.	.	S	–	–	–	–	–
A/equine/Wisconsin/1/03	.	.	.	.	.	.	.	K	I	.	.	.	.	.	.	S	E	K	P	K	I
A/equine/California/1/2007	.	.	.	.	.	.	.	K	I	.	.	.	.	.	.	S	–	–	–	–	–
A/equine/Cheshire/1/2006	.	.	.	.	.	.	F	K	I	.	.	.	.	S	.	.	E	.	P	.	.
A/equine/Kentucky/1/1992	.	.	.	.	.	.	.	K	I	.	.	.	.	.	.	.	E	.	P	.	.
A/equine/Newmarket/1/1993	.	.	.	.	.	.	F	K	I	.	.	.	.	S	.	.	E	.	P	.	.
A/equine/Newmarket/5/2003	.	.	.	.	.	.	.	K	I	.	.	.	.	.	.	S	–	–	–	–	–

Accessions numbers: Moroccan strains: A/equine/Nador/1/1997, *AFN69204*; A/equine/Essaouira/2/2004, *AFJ69907*; A/equine/Essaouira/3/2004, *AFJ69911*. Predivergent strains: A/equine/Miami/1/1963, *ABY81497*; A/equine/Uruguay/1/1963, *ACD85423*; A/equine/Algiers/1/1972, *ACF22131*; A/equine/Romania/1/1980, *ACD85379*; A/equine/Fontainebleau/1979, *AAC35571*. European lineage: A/equine 2/Suffolk/89, *CAA56366*; A/equine/Yvelines/2136/1989, *AAC31256*; A/equine/Newmarket/2/1993, *ACI48782*; A/equine/Italy/1199/1992, *ACD85313*; A/equine/Rome/5/1991, *AAC31262*; A/equine/Avesta/1/1993, *BAI67724*; A/equine/Berlin/1/1989, *ACD85412*; A/equine/Grobois/1/1998, *AAP92165*. American lineage: A/eq/LaPlata/93, *AAC31270*; A/equine/Florida/2/2006, *ACH95625*; A/equine/Ohio/1/2003, *ABA42431*; A/equine/Richmond/1/2007, *ACH95617*; A/equine/Kentucky/7/2007, *ACH95629*; A/equine/Wisconsin/1/03, *ABB17179*;.A/equine/California/1/2007, *ACH95621*; A/equine/Cheshire/1/2006, *ACH95605*; A/equine/Kentucky/1/1992, *ACA24639*; A/equine/Newmarket/1/1993, *ACI48792*; A/equine/Newmarket/5/2003, *ACI48802*

., No mutation compared to the reference strain; —, Amino acid residue is not available

The NS1 protein of both strains of Essaouira presents an identical sequence of them. Therefore, compared to reference strain A/equine/Miami/1/1963, the carboxyl terminus of the NS1 proteins shows a common mutations regarding substitution S/228/P. Moreover, the PDZ-binding sequence, Glu-Pro-Glu-Val (EPEV) motif appears in both avian as well as swine, human and equine viruses.

In addition, the presence of the EPEV sequence in the NS1 protein of three equine Moroccan strains is an indicator of virulence [30]. This sequence, present at the C-terminus of NS1 of the H5N1 virus (sequence ESEV/EPEV) and the H1N1 pandemic of 1918 (sequence KSEV) but absent among seasonal human viruses, establishes binding with the PDZ domains [31]. The ESEV sequence is common among avian viruses. Its presence in NS1 of the A/equine/Miami/1/1963(H3N8) strain is related to its ancestral avian virus, which infected horses of North America in 1963 [32], while the S/228/P mutation observed in the three Moroccan strains is considered to be an adaptation of avian influenza viruses (H3N8) origin to the new equine host [33].

## Conclusion

The study of NS1 sequences of three Moroccan strains shows that all mutations are not produced at the key residues of RBD (RNA-binding domain) and ED (effector domain) domains, and therefore do not appear to affect the potency of inhibition of cellular factor defences, and their virulence.

## Abbreviations

BLAST: basic local alignment search tool
RT-PCR: reverse transcriptase-polymerase chain reaction
RBD: RNA-binding domain
ED: effector domain
NS: non-structural protein
NEP: nuclear export protein
